# Sex-Related Differences in Patients With Unexplained Syncope and Bundle Branch Block: Lower Risk of AV Block and Lesser Need for Cardiac Pacing in Women

**DOI:** 10.3389/fcvm.2022.838473

**Published:** 2022-02-25

**Authors:** Jaume Francisco-Pascual, Nuria Rivas-Gándara, Montserrat Bach-Oller, Clara Badia-Molins, Manel Maymi-Ballesteros, Begoña Benito, Jordi Pérez-Rodon, Alba Santos-Ortega, Antonia Sambola-Ayala, Ivo Roca-Luque, Javier Cantalapiedra-Romero, Jesús Rodríguez-Silva, Gabriel Pascual-González, Àngel Moya-Mitjans, Ignacio Ferreira-González

**Affiliations:** ^1^Arrhythmia Unit, Cardiology Department, Hospital Universitari Vall d'Hebron, Vall d'Hebron Institut de Recerca (VHIR), Vall d'Hebron Barcelona Hospital Campus, Barcelona, Spain; ^2^Department of Medicine, Universitat Autònoma de Barcelona, Bellaterra, Spain; ^3^CIBER de Enfermedades Cardiovasculares (CIBERCV), Instituto de Salud Carlos III, Madrid, Spain; ^4^Cardiology Department, Hospital Universitari Vall d'Hebron, Vall d'Hebron Institut de Recerca (VHIR), Vall d'Hebron Barcelona Hospital Campus, Barcelona, Spain; ^5^Arrhythmia Section, Institut Clinic Cardiovascular, Hospital Clínic, Barcelona, Spain; ^6^Cardiology Department, Hospital Universitari Dexeus, Barcelona, Spain; ^7^CIBER de Epidemiología y Salud Pública (CIBERESP), Instituto de Salud Carlos III, Madrid, Spain

**Keywords:** syncope, pacemaker, electrophysiological study, loop recorder, cardiac monitor, gender differences, sex-related differences

## Abstract

**Objective:**

To analyze if there are sex-related differences in patients with unexplained syncope and bundle branch block (BBB).

**Background:**

Despite increasing awareness that sex is a major determinant of the incidence, etiology, and the outcomes of different arrhythmias, no studies have examined differences in presentation and outcomes between men and women with syncope and BBB.

**Methods:**

Cohort study of consecutive patients with unexplained syncope and BBB was included from January 2010 to January 2021 with a median follow-up time of 3.4 years [interquartile range (IQR) 1.7–6.0 years]. They were evaluated by a stepwise workup protocol based on electrophysiological study (EPS) and long-term follow-up with an implantable cardiac monitor (ICM).

**Results:**

Of the 443 patients included in the study, 165 (37.2%) were women. Compared with men, women had less diabetes (25.5 vs. 39.9%, *p* = 0.002) and less history of ischemic heart disease (IHD; 13.3 vs. 25.9%, *p* = 0.002). Left bundle branch block (LBBB) was more frequent in women (55.2 vs. 27.7%, *p* < 0.001) while right bundle branch block (RBBB) was more frequent in men (41.5 vs. 67.7%, *p* < 0.001). His to ventricle (HV) interval in the EPS was shorter in women (58 ms [IQR 52–71] vs. 60 ms [IQR 52–73], *p* = 0.035) and less women had an HV interval longer than 70 ms (28.5 vs. 38.1%, *p* = 0.039), however, EPS and ICM offered a similar diagnostic yield in both sexes (40.6 vs. 48.9% and 48.4% vs. 51.1%, respectively). Women had a lower risk of developing atrioventricular block (AVB) (adjusted odds ratio [OR] 0.44–95% CI 0.26–0.74, *p* = 0.002) and of requiring permanent pacemaker implantation (adjusted hazard ratio [HR] 0.72–95% CI: 0.52–0.99, *p* = 0.046). The mortality rate was lower in women (4.5 per 100 person-years [95% CI 3.1–6.4 per 100 person-years] vs. 7.3 per 100 person-years [95% CI 5.9–9.1 per 100 person-years]).

**Conclusions:**

Compared to men, women with unexplained syncope and BBB have a lower risk of AVB and of requiring cardiac pacing. A stepwise diagnostic approach has a similar diagnostic yield in both sexes, and it seems appropriate to guide the treatment and avoid unnecessary pacemaker implantation, especially in women.

## Graphical Abstract

Risk of AVB and need for cardiac pacing. Left: Percentage of patients diagnosed with aAVB/sCD in both sexes. Right-top: Multivariate logistic regression analyses for risk of aAVB/sCD. Odds ratio and 95% CI are plotted. Right-bottom: Kaplan-Meier pacemaker-free survival estimates curves in both sexes. aAVB/sCD, advanced atrio-ventricular block or severe conduction disturbances; HR, hazard ratio; CI, confidence interval; y.o, years old; IHD, ischemic heart disease; LVEF, left ventricular ejection fraction; BBB, bundle branch block; LBBB, left bundle branch block; RBBB, right bundle branch block; LAFB, left anterior fascicular block.
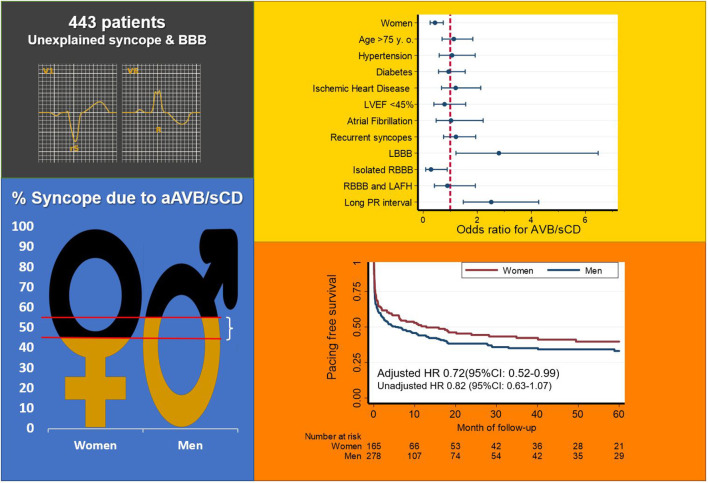

## Introduction

Although syncope in patients with bundle branch block (BBB) is often due to paroxysmal advanced atrioventricular block (aAVB), other mechanisms may also be involved (1–4). A systematic diagnostic approach based on clinical evaluation, electrophysiological study (EPS), and the Implantable cardiac monitor (ICM) has shown to be safe and provide a high rate of etiological diagnosis (3, 5–7). However, due to the low predictive value of EPS, some investigators suggest that a pacemaker should be implanted on an empirical basis (2, 8), therefore, the best way to manage these patients remains controversial. Increasing knowledge of the disease characteristics can help clinicians to improve their management in specific subgroups of patients. Despite substantial efforts in recent years to improve the understanding of the sex-related differences in cardiovascular disease, there is still insufficient knowledge of physiology, epidemiology, and outcomes in women, leading to a lack of sex-specific recommendations. In this regard, there is an increasing awareness that sex is a major determinant of the incidence, etiology, and clinical presentation of arrhythmias (9, 10). It is known that women have a major susceptibility to reflex syncope (11–14) and probably to sinus node dysfunction (SND) (9, 10, 15). However, no studies have examined differences between men and women in the presentation and outcomes of unexplained syncope and BBB.

Given the susceptibility of women to syncope due to other mechanisms and the different comorbidities of the female sex, we hypothesize that women with unexplained syncope and BBB would have a different risk of aAVB or severe conduction disturbances (sCDs) and a different risk of needing cardiac pacing compared to men. The aim of this study was to analyze the sex-related differences in patients with syncope and BBB concerning the prevalence of aAVB/sCD, the diagnostic yield of tests, and clinical outcomes.

## Methods

### Study Population

We performed a prospective observational study on a consecutive patient cohort at a tertiary university hospital that is a reference center for cardiology and arrhythmias [Hospital Universitari Vall d'Hebron, Barcelona (Spain)]. From January 2010 to January 2021, we included those patients admitted for syncope with BBB, in whom no certain diagnosis was reached for the syncope in the initial assessment at the emergency department. We excluded patients under the age of 18 years, those with pacemakers or implantable cardiac defibrillators (ICD) *in situ*, patients with left ventricular ejection fraction (LVEF) <35% or with another ICD direct indication, and those who could not keep to the study's diagnostic protocol due to comorbidities or their own preference. In June 2021, we collected the final follow-up data of the patients. The patient's clinical details, syncope characteristics, therapeutic management, and follow-up were recorded at the time of hospital admission.

The study complies with the Helsinki declaration and was approved by the local ethics committee.

### Study Protocol

Patients were systematically assessed and managed according to the local clinical protocol which is based on recommendations from the European Society of Cardiology (ESC) syncope guidelines (1).

**Figure 1:**
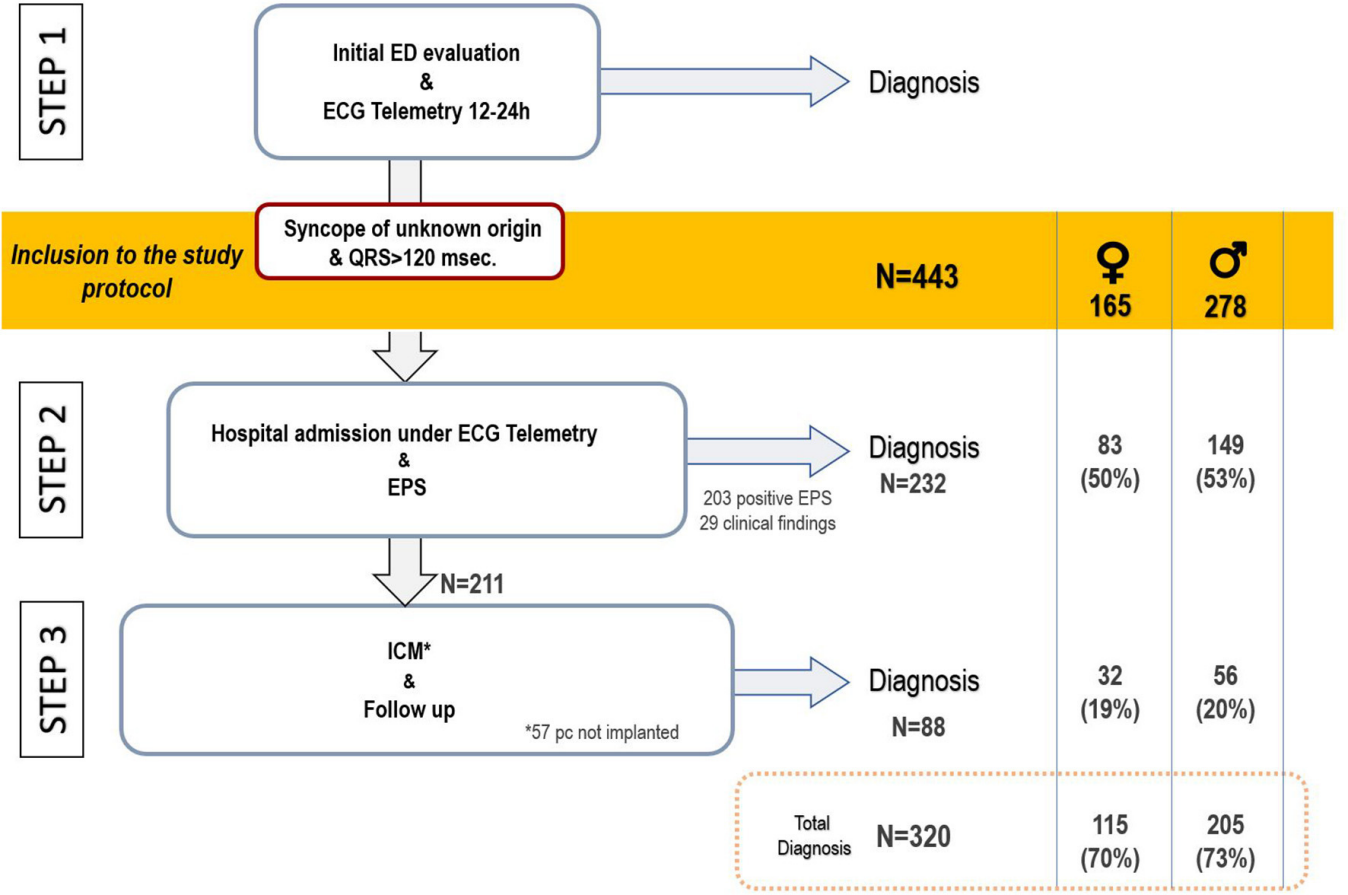
Diagnostic protocol schema and flow chart of patient inclusion in the study. ED, emergency department; EPS, electrophysiological study; ICM, implantable cardiac monitor; pc, patients.

In summary, the diagnostic protocol for syncope in this population was based on 3 phases or steps. Step 1, prior to the patients' inclusion in the study, consisted of the initial assessment in the emergency department. In a systematic manner, clinical history and physical examination were performed, such as testing for orthostatic hypotension and carotid sinus massage (if not contraindicated), general bloodwork, chest x-ray, 12-lead ECG, 12–24-h telemetry monitoring and a transthoracic echocardiogram (in cases where no prior echocardiogram from the last 6 months is available). Those cases with no certain or highly probable diagnosis were then considered unexplained syncope, and these patients were admitted to the hospital with continuous ECG monitoring. Other complementary diagnostic tests, such as exercise stress test, myocardial perfusion gamma scan, or MRI, were carried out at the treating clinician's discretion in line with the suspected diagnosis and applicable recommendations. Step 2 involved the hospital admission with continuous ECG monitoring and an invasive electrophysiology study. Step 3 involved implanting an ICM with subsequent clinical monitoring (Figure 1).

### Electrophysiology Study

Two femoral venous accesses were gained and two tetrapolar catheters (Supreme, Abbott, St. Jude Medical, St. Paul, MN, USA) were used for basic measurements, atrial stimulation, and ventricular stimulation. Sinus node recovery time was obtained after 30 s of atrial pacing at 600 and 500 ms, and the highest value was corrected by basal heart rate. Programmed ventricular stimulation protocol utilized up to three extra stimuli delivered after eight paced ventricular cycle lengths at 600, 500, and 400 ms from de right ventricular apex and outflow tract in case no sustained ventricular tachycardia (VT) was induced before.

In cases with basal conduction disturbances where the His to ventricle (HV) interval was <70 ms, a class I drug (procainamide 10 mg/kg or flecainide 2 mg/kg intravenously) was administered. Continuous monitoring of the HV interval and atrial pacing was performed during the class I drug infusion and for 10 min after the infusion.

Electrophysiological study was considered positive according to current ESC guidelines (1) in the following cases: (1) baseline HV interval ≥70 ms or ≥100 ms after class I drug administration. (2) Second- or third-degree infra- or intra-Hisian block (with pacing cycle length above 400 ms) before or during incremental atrial pacing or after class I drug administration. (3) Induction of sustained VT.

### Monitoring With Implantable Cardiac Monitor

In Step 3, a Reveal XT^TM^ (in patients included before 2014) or Linq^TM^ (Medtronic, Inc. Minneapolis, MN, USA) device was implanted. The implantation was performed under local anesthetic at the primary site recommended by the manufacturer (fourth left intercostal space). The patients were instructed on how to use it and were provided with a device for remote monitoring (Medtronic Carelink^TM^). The ICM was programmed with the settings for syncope.

Implantable cardiac monitor was considered diagnostic in the event of being able to correlate recurrence of syncope or presyncope with the ICM's electrocardiographic trace, or when the following rhythm disorders were documented in an asymptomatic patient: complete or advanced AV block, asystole lasting >3 s while awake, or the presence of sustained VT.

### Treatment and Clinical Follow-Up

The syncope was treated appropriately following the clinical practice guidelines according to its etiology. In those patients with syncope secondary to a conduction disorder, the implantation of a cardiac stimulation device was indicated. In patients with syncope secondary to ventricular tachycardia, defibrillator implantation was indicated. The device type (pacemaker, defibrillator, or resynchronizer) and treatments, such as ablation, antiarrhythmic drugs, or angioplasty, were eventually discussed within the “heart team” and individualized according to the patient's functional status, the prior degree of heart failure, and patient preferences. In addition, all patients were educated on syncope and lifestyle changes to prevent and treat reflex syncope.

After hospital discharge, patients were followed up in the outpatient cardiology clinic, and those who had received a cardiac device were also followed up with the corresponding remote function.

### Definitions and Endpoints

The main etiological mechanism of the syncope was established as certain or highly probable according to the definitions in the ESC guidelines on syncope (1) (Supplementary Table S1). aAVB/sCD was defined as the documentation of type II second degree, third degree, or high-grade AVB or the following diagnostic findings in the EPS: HV interval ≥ 70 ms or ≥100 ms after class I drug challenge, intra-Hisian, or infra-Hisian block (1, 16). The patient details were analyzed by two cardiologists specialized in syncope to establish the definitive diagnosis according to the definitions. The etiology of syncopal recurrences was defined in the same manner.

Sudden death was defined as death occurring instantaneously or within 1 h of the onset of symptoms, non-sudden cardiac death was defined as a cardiac death occurring 1 h after the onset of symptoms, and non-cardiac death as deaths not directly related to a cardiac or sudden condition.

The primary endpoint of the study was a diagnosis of the main syncope mechanism. The secondary endpoints were test diagnostic yields, need for cardiac pacing related to syncope, syncope recurrences, and mortality.

### Statistical Analysis

The categorical variables are presented as absolute number (N) and percentages. The continuous quantitative variables are presented as the median and interquartile range (IQR). The comparison of numerical variables was performed using Student's *t*-test or Wilcoxon's rank-sum test, depending on the distribution of the variables. The Chi-squared test or Fisher's exact test was used to compare qualitative variables as appropriate. Wald's method was used to calculate the CI for the population rates and proportions. The survival functions were estimated using the Kaplan-Meier method and their comparison was performed by the log-rank test. A multivariable logistic regression model was used to assess the association between sex and aAVB/sCD and to adjust for possible confounder variables. Moreover, a Cox proportional hazards multivariate model was created to determine whether the sex was associated with pacemaker implantation adjusted by possible confounding variables. When we estimated both the Cox proportional hazards model and the logistic regression model, we checked the different possible interactions between pairs of explanatory variables and found no statistically significant results. A saturated model, such as all clinically relevant covariates (1, 4, 5, 7, 17–22), was estimated, and simplified models were evaluated. A relevant confounding effect was judged when the hazard ratios (HRs) or odds ratios (ORs) with and without the adjustment for the potential confounder differed more than 10%. The most precise model with all relevant clinical covariates was finally selected. A *p* < 0.05 was considered statistically significant for all tests. All of the statistical analyses were performed using Stata, version 15.1.0 (StataCorp LLC College Station, TX, USA).

## Results

### Baseline Characteristics

A total of 443 patients were included in the study, of whom 165 (37.2%) were women. The patients' baseline characteristics and the comparisons between men and women are shown in Table 1. The median age was 77.9 years [IQR 70.5–82.1] and 21.2% had ischemic heart disease (IHD). The median LVEF was 58% [IQR 51–62%] and 14.7% of the patients had a depressed LVEF (<45%). The median QRS duration was 140 ms [IQR 130–153 ms]. In the ECG on admission, 37.9% of patients had typical left bundle branch block (LBBB) morphology and 58.6% right bundle branch block (RBBB) morphology.

**Table 1:** Baseline characteristics of patients included in the study.

**Variable**	**Total (n = 443)**	**Men (n = 278)**	**Women (n = 165)**	**P**
Age (years)^+^	77.9 [70.5–82.1]	77.0 [70.3–82.20]	78.7 [71.2–84.6]	0.122
Age >75 y.o, *n* (%)	273 (61.6)	167 (60.1)	106 (64.2)	0.383
Hypertension, *n* (%)	348 (78.6)	223 (80.2)	125 (75.8)	0.269
Diabetes, *n* (%)	153 (34.5)	111 (39.9)	42 (25.5)	0.002
Dyslipidemia, *n* (%)	266 (60.1)	168 (60.4)	98 (59.4)	0.829
No SHD, *n* (%)	346 (78.1)	212 (76.3)	134 (81.2)	0.223
Ischemic heart disease, *n* (%)	94 (21.2)	72 (25.9)	11 (13.3)	0.002
Old ST elevation infarction, *n* (%)	25 (5.6)	20 (7.2)	5 (3.0)	0.066
Non-ischemic dilated cardiomyopathy, *n* (%)	16 (3.6)	9 (3.2)	7 (4.2)	0.584
History of atrial fibrillation, *n* (%)	90 (20.3)	62 (22.3)	28 (17.0)	0.177
Previous syncope, *n* (%)	235 (53.1)	154 (55.4)	81 (49.1)	0.199
Use of negative chronotropic drugs, *n* (%)	149 (34.8)	95 (35.3)	54 (34.0)	0.776
**Characteristics of the syncope**				
Prodrome, *n* (%)	134 (30.5)	84 (30.3)	50 (30.8)	0.776
Severe trauma, *n* (%)	185 (42.1)	121 (43.6)	64 (39.5)	0.393
**Echocardiogram**				
EDD (mm)	47 [43–52]	48 [43–53]	46 [42–50]	<0.001
ESD (mm)	31 [26–35]	32 [27–36]	30 [26–34]	0.016
Interventricular septum (mm)	13 [11–14]	13 [12–14]	12 [10–15]	0.021
LVEF (%)	58 [51–62]	57 [50–62]	58 [52–62]	0.746
LVEF <45%, *n* (%)	61 (14.7)	38 (14.8)	23 (14.7)	0.970
**ECG on admission**				
Heart rate (bpm)	70 [62–80]	70 [60–80]	70 [63–80]	0.996
Atrial fibrillation, *n* (%)	78 (17.8)	49 (17.9)	29 (17.6)	0.935
Long PR, *n* (%)	152 (40.2)	104 (43.7)	48 (34.3)	0.720
QRS duration (msec)	140 [130–153]	140 [130–153]	140 [130–152]	0.891
LBBB morphology, *n* (%)	167 (37.9)	77 (27.7)	90 (55.2)	<0.001
Long PR and LBBB, *n* (%)	47 (10.6)	24 (8.6)	23 (13.9)	0.080
RBBB morphology, *n* (%)	259 (58.6)	191 (67.7)	68 (41.5)	<0.001
Isolated RBBB	50 (11.7)	34 (12.6)	16 (10.2)	0.449
RBBB and LAFB	159 (35.9)	116 (41.7)	43 (26.1)	0.001
Long PR and RBBB	96 (21.7)	75 (27.)	21 12.7)	<0.001
Long PR, RBBB and LAFB	71 (16.0)	52 (18.7)	19 (11.5)	0.046

+ *The quantitative variables are expressed as medians [interquartile range]*.

*y.o, years old; mm, millimeters; bpm, beats per minute; msec, milliseconds; SHD, structural heart disease; LBBB, left bundle branch block; RBBB, right bundle branch block; LAFB, left anterior fascicular block. ESD, end-systolic diameter; EDD, end-diastolic diameter; LVEF, left ventricular ejection fraction*.

Compared with men, women had less diabetes (25.5 vs. 39.9%, *p* = 0.002) and less history of IHD (13.3 vs. 25.9%, *p* = 0.002). However, there were no differences regarding atrial fibrillation history and other comorbidities. The rate of conduction disturbances in the ECG on admission differed between both sexes: LBBB was more frequent in women (55.2 vs. 27.7%, *p* < 0.001) while RBBB was more frequent in men (41.5 vs. 67.7%, *p* < 0.001).

### Etiology of the Syncope and Risk of aAVB/sCD

A certain or highly probable diagnosis of the main cause of syncope was reached in 320 patients (72.2%). In 232 (52.4%) patients, the diagnosis of syncope was reached in Step 2 (in 203 patients after a positive EPS and in another 29 due to presenting symptoms with diagnostic criteria during hospital stay). In Step 3, a definitive diagnosis was reached in an additional 88 (19.9%) patients (77 due to the ICM findings and 11 due to clinical criteria; Figure 1).

Table 2 summarizes the etiologies of syncope and the diagnoses reached in each step. Compared to men, women had less frequent aAVB/sCD (44.9 vs. 55.0%, *p* = 0.038), which represents a risk ratio (RR) of 0.81 (95% CI 0.67–0.99). Furthermore, in multivariate analyses, after adjusting for possible confounding variables (such as the type of BBB), women had a lower risk of developing aAVB/sCD than men [OR 0.44 (95% CI 0.26–0.74, *p* = 0.002); Graphical Abstract and Supplementary Table S2].

**Table 2:** Etiological diagnosis.

**Diagnostic**	**Total**				**Step 2**				**Step 3**			
	**All patients**	**Men**	**Women**	**P**	**All patients**	**Men**	**Women**	**P**	**All patients**	**Men**	**Women**	**P**
	**(n = 443)**	**(n = 278)**	**(n = 165)**		**(n = 443)**	**(n = 278)**	**(n = 165)**		**(n = 211)**	**(n = 129)**	**(n = 82)**	
Unknown, *n* %	123 (27.8)	73 (26.3)	50 (30.3)	0.358	211 (47.6)	129 (46.4)	82 (49.7)	0.502	123 (58.3)	73 (56.6)	50 (61.0)	0.529
aAVB/sCD, *n* %	227 (51.2)	153(55.0)	74 (44.9)	0.038	194 (43.8)	120(46.8)	64 (38.8)	0.045	33 (15.6)	23 (17.8)	10 (12.2)	0.272
Orthostatic, *n* %	31 (7.0)	21 (7.6)	10 (6.1)	0.551	10 (2.3)	5 (1.8)	5 (3.3)	0.337	21 (10.0)	16 (12.4)	5 (6.1)	0.136
SND, *n* %	22 (5.0)	11 (4.0)	11 (6.7)	0.204	3 (0.7)	2 (0.7)	1 (0.6)	>0.999	19 (9.0)	9(7.0)	10 (12.2)	0.197
Reflex, *n* %	15 (3.4)	8 (2.9)	7 (4.2)	0.443	8 (1.8)	5 (1.8)	3 (1.8)	>0.999	7 (3.3)	3 (2.3)	4 (4.9)	0.435
Low cardiac output, *n* %	5 (1.1)	1 (0.4)	4(2.4)		5 (1.4)	1 (0.4)	4 (2.4)		0 (0)	0 (0)	0 (0)	
VT, *n* %	6 (1.4)	2 (0.4)	4 (1.2)		5 (1.1)	2 (0.7)	3 (1.8)		1 (0.5)	0 (0)	1 (1.2)	
Fast SVT/AF, *n* %	3 (0.7)	1 (0.4)	2 (1.2)		1 (0.2)	0 (0)	1 (0.6)		2 (1.0)	1 (0.8)	1 (1.2)	
CSH, *n* %	3 (0.7)	3 (1.1)	0 (0)		3 (0.7)	2 (0.7)	1 (0.6)		0 (0)	0 (0)	0 (0)	
Other, *n* %	8 (1.8)	5 (1.8)	3 (1.8)		3 (0.7)	1 (0.4)	2 (1.2)		5 (2.4)	4 (3.1)	1 (1.2)	

*aAVB/sCD, advanced atrio-ventricular block or severe conduction disturbances; VT, ventricular tachycardia; SND, sinus node dysfunction; SVT, supraventricular tachycardia; AF, atrial tachycardia; CSH, carotid sinushypersensitivity*.

### EPS and Implantable Cardiac Monitor

Overall, EPS was positive in 203 (45.8%) patients, and it was due to aAVB/sCD in 193 (43.6%). Details of the EPS results are listed in Table 3. Baseline HV interval was shorter in women (58 ms [IQR 52–71] vs. 60 ms [IQR 52–73], *p* = 0.035) than in men. Furthermore, fewer women had a baseline HV interval longer than 70 ms (28.5% vs. 38.1%, *p* = 0.039).

**Table 3:** Electrophysiological study and implantable cardiac monitor.

**Variable**	**Total**	**Men**	**Women**	**P**
	**(*n* = 443)**	**(*n* = 278)**	**(*n* = 165)**	
**Electrophysiological study**				
Baseline HV interval (msec)	59 [52–73]	60 [52–73]	58 [52–71]	0.035
HV≥70, *n* (%)	153 (34.5)	106 (38.1)	47 (28.5)	0.039
Intra or infra-Hisian AV block, *n* (%)	30 (6.9)	14 (5.1)	16 (9.9)	0.06
Basal EPS positive for aAVB/sCD, *n* (%)	168 (37.9)	112 (40.3)	56 (33.9)	0.183
Class I drug challenge, *n* %	241 (55.1)	146 (53.1)	95 (58.6)	0.349
Procainamide, *n* %	93 (21.3)	59 (21.2)	34 (21.0)	
Flecainide, *n* %	147 (33.6)	87 (31.6)	60 (37.0)	
HV interval after class I challenge (msec)	69 [61-78]	69 [61-78]	71 [61-78]	0.689
Delta HV interval (msec)	15 [10–22]	15 [10–22]	15 [11–21]	0.77
HV≥100 after class I challenge, *n* (%)	14 (3.2)	11 (3.4)	3 (1.8)	0.27
Intra or infra-Hisian AV block after IC challenge, *n* (%)	15 (6.0)	10 (6.4)	5 (5.4)	0.749
Positive class I challenge, *n* (%)	25 (10.3)	17 (11.6)	8(8.4)	0.433
cSNRT (msec)	210 [153–280]	206 [150–278]	220 [160–294]	0.492
VT induction, *n* (%)	6 (3.6)	2 (1.9)	4 (6.1)	0.211
EPS positive for aAVB/sCD, *n* (%)	193 (43.6)	129 (46.4)	64 (37.8)	0.118
EPS positive for all diagnoses, *n* (%)	203 (45.8)	136 (48.9)	67 (40.6)	0.089
**Implantable cardiac monitor**				
**Patients implanted**	***n*** **= 154**	***n*** **= 92**	***n*** **= 62**	
ICM diagnostic, *n* (%)	77 (50)	47 (51.1)	30 (48.4)	0.742
Asymptomatic finding, *n* (%)^*^	23 (29.9)	14 (29.8)	9 (30.0)	0.984
Symptomatic finding, *n* (%)^*^	54 (70.1)	33 (70.2)	21 (70.0)	

* *% refers to the total of patients diagnosed by ICM*.

*HV, His to ventricle; aAVB/sCD, advanced atrio-ventricular block or severe conduction disturbances; VT, ventricular tachycardia; EPS, electrophysiological study; ICM, implantable cardiac monitor; cSNRT, corrected sinus node recovery time; msec, milliseconds*.

**Figure 2:**
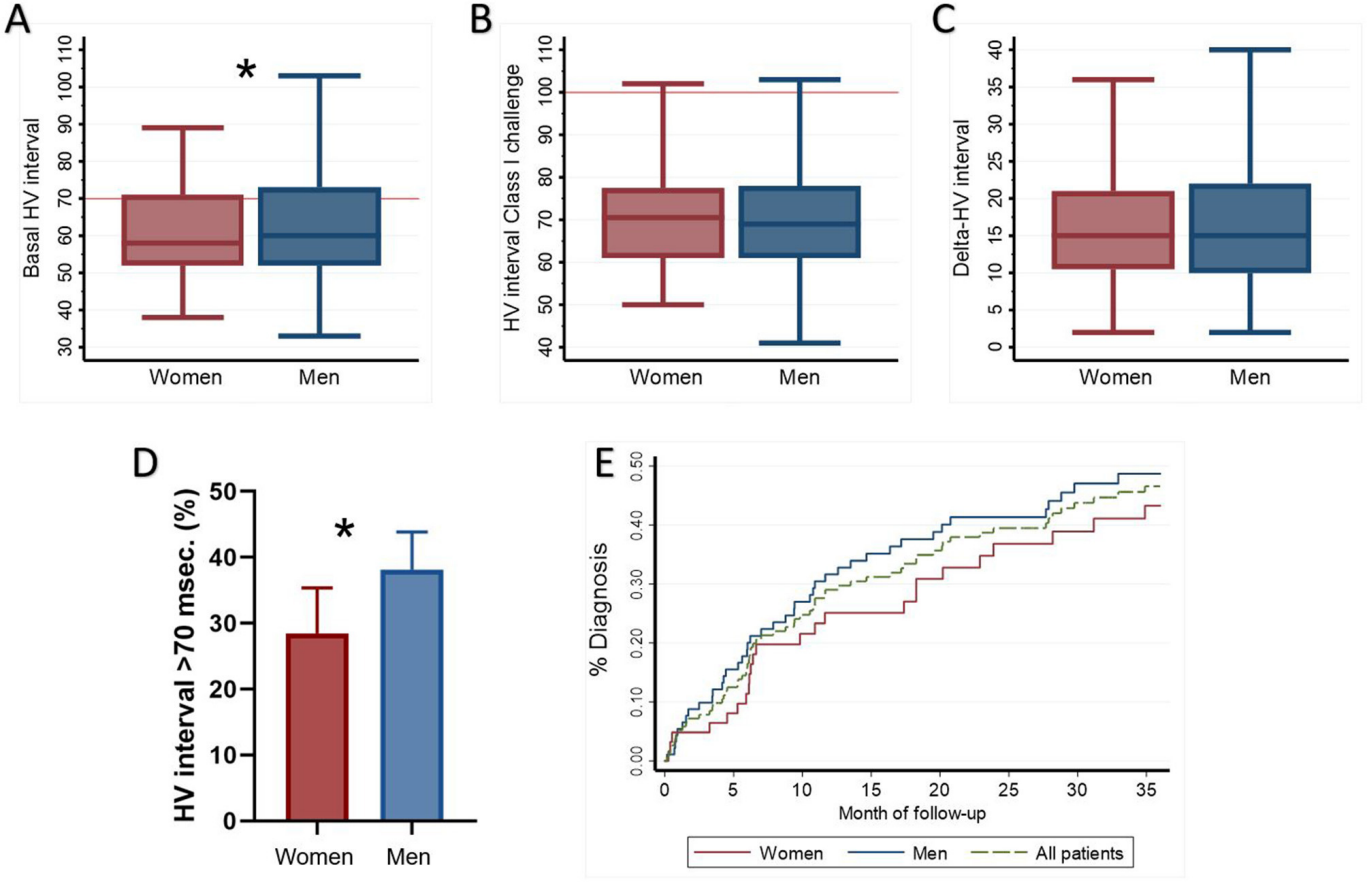
Electrophysiological study and implantable cardiac monitor. A comparison between sex of **(A)** baseline HV interval, **(B)** HV interval after class I challenge, and **(C)** absolute increase of HV interval (Delta HV) after class I challenge. **(D)** Percentage of patients with basal HV interval >70 ms in EPS. **(E)** ICM cumulative diagnostic yield according to time of follow-up. ^*^p < 0.05. HV, His-ventricular; ICM, implantable cardiac monitor.

Among those patients with negative EPS at baseline [241 patients (55.1%)], class I drug challenge was positive in 25 (10.3%). No significant differences between men and women were found in the increase of HV interval (Delta HV) or in the positivity of the test (Figure 2).

Electrophysiological study had a similar diagnostic yield between women and men (40.6 vs. 48.9%, *p* = 0.089). In addition, EPS negative predictive value (NPV) was similar between both sexes (76.6% [95% CI 67.1–84.0%] vs. 76.6 % [95% CI 69.0–82.8%]).

Among 154 patients who received an ICM, in 77 patients (50% of the implanted patients) a diagnosis was reached, with a similar diagnostic yield between both sexes (48.4% in women and 51.1% in men, *p* = 0.742; Table 3 and Figure 2).

### Pacemaker Implantation, Clinical Follow-Up, and Prognosis

**Figure 3:**
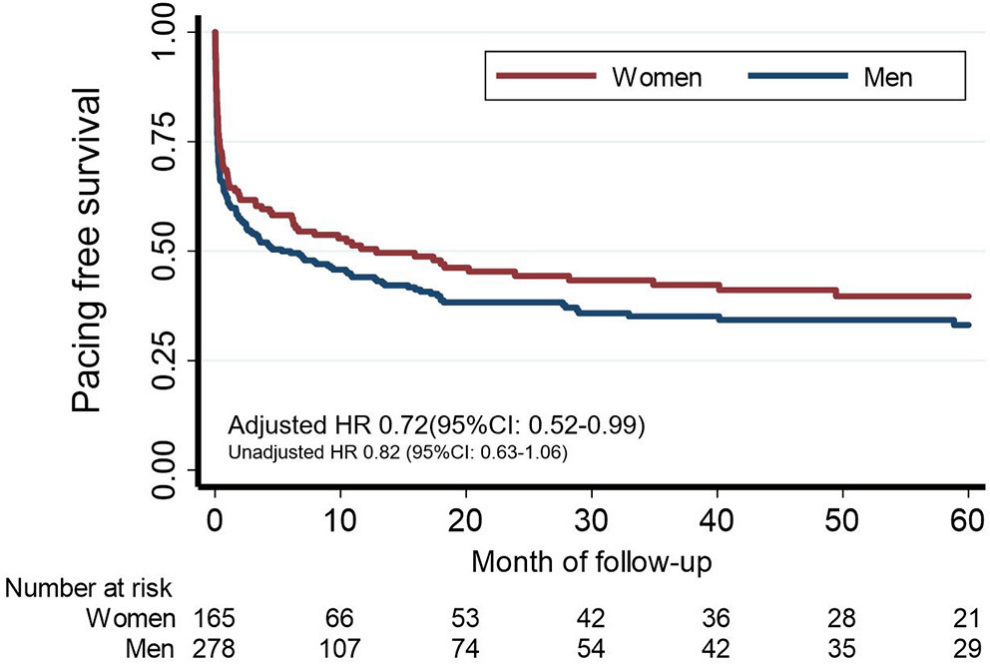
Cardiac pacing. Kaplan-Meier pacemaker-free survival estimates curves for women and men.

Patients were followed for a median of 3.4 years [IQR 1.7–6.0 years]. A total of 252 (58.2%) patients required pacing due to bradycardia related to the syncope at the end of follow-up (Table 4; Supplementary Table S3 shows the type of device implanted). Additionally, 2 ICD and 2 CRT-D were implanted due to ventricular tachycardia, 3 pacemakers due to post-surgical AV block, and 3 additional pacemakers because of chronotropic insufficiency. Two patients with VT were treated with antiarrhythmic drugs only due to their comorbidities. In a Cox multivariate analysis, after adjusting for possible confounding variables, women had a lower risk of needing permanent pacemaker implantation compared to men [adjusted HR 0.72 (95% CI: 0.52–0.99, *p* = 0.046); Table 5 and Figure 3].

**Table 4:** Outcomes during follow-up.

**Variable**	**Total**	**Men**	**Women**	***P***
	**(*n* = 443)**	**(*n* = 278)**	**(*n* = 165)**	
Median follow-up time (years)	3.4 [1.7–6.0]	3.4 [1.5–5.8]	3.2 [1.8–6.2]	0.845
**Pacing requirements**				
Total patients requiring pacing due to the syncope, *n* (%)	252 (58.2)	167 (60.7)	85 (53.8)	0.159
Devices implanted during admission, *n* (%)	198 (44.7)	134 (48.2)	64 (38.8)	0.054
Devices implanted during follow up, *n* (%)	54 (22.5)	33 (23.24)	21 (21.4)	0.741
**Syncope recurrence**				
Total syncope recurrence, *n* (%)	95 (21.4)	63 (22.7)	32 (19.4)	0.418
Syncope recurrence after diagnosis, *n* (%)	30 (8.9)	19 (8.9)	11 (8.9)	0.998
**Mortality**				
Total deaths, *n* (%)	111 (25.1)	81 (29.1)	30 (18.2)	0.010
Mortality rate, (x100 person-years)	6.3	7.3	4.5	0.009
Cause of death				
Cardiovascular death	26 (23.4)	18 (22.2)	8 (26.7)	0.686
Non-cardiovascular death	81 (73.0)	60 (74.1)	21 (70.0)	
Unknown	4 (3.6)	3 (3.7)	1 (3.3)	

**Table 5:** Cox proportional hazards multivariate model to assess the association between sex and pacing needs.

	**Factor**	**HR**	**HR 95% CI**	***p*** **-value**
Unadjusted				
	**Women**	**0.82**	**0.63–1.06**	**0.131**
Adjusted				
	**Women**	**0.72**	**0.52–0.99**	**0.046**
	Age>75 y.o	1.19	0.89–1.61	0.247
	Hypertension	1.06	0.73–1.54	0.765
	Diabetes	1.09	0.80–1.48	0.586
	IHD	1.22	0.86–1.75	0.266
	LVEF <45%	0.87	0.56–1.35	0.542
	Atrial fibrillation	1.09	0.70–1.70	0.698
	Recurrent syncope	1.20	0.89–1.61	0.236
	LBBB	1.54	0.95–2.50	0.080
	Isolated RBBB	0.30	0.12–0.68	0.005
	RBBB and LAFH	1.05	0.65–1.69	0.846
	Long PR interval	1.62	1.20–2.19	0.002

*CI, confidence interval; HR, hazard ratio; y.o, years old; IHD, ischemic heart disease; LVEF, left ventricular ejection fraction; LBBB, left bundle branch block; RBBB, right bundle branch block; LAFB, left anterior fascicular block*.

After the etiological diagnosis and appropriate treatment, 30 patients (8.9%) experienced a syncopal recurrence (Table 4), most of them due to a vagal or orthostatic mechanism (Supplementary Table S4).

A total of 111 (25.1%) patients died during the follow-up, 73% of them due to non-cardiovascular causes. Only 2 patients experienced sudden death, one 80 years old female with syncope of unknown origin and one 79 years old male with a pacemaker implanted due to AVB 4 years before. The mortality rate in women was 4.5 per 100 person-years (95% CI 3.1–6.4 per 100 person-years) and 7.3 per 100 person-years (95% CI 5.9–9.1 per 100 person-years) in men.

## Discussion

As far as we know, this is the first cohort study to specifically evaluate sex-related differences in patients with unexplained syncope and BBB. In addition, it is one of the largest patient cohorts published evaluating the etiology of syncope and outcomes in this population. The main findings of this study are that women with syncope of unknown origin and BBB are at lower risk of having aAVB/sCD and of requiring pacemaker implantation than men.

In the general population, syncope seems to be more frequent in women (1, 2, 14, 21, 23). In a recent national population-based cohort study that included more than 2.5 million participants, Fedorowsky et al. (21) found that 62% of the patients with syncope were women. However, this proportion is reversed when a cohort of patients with structural heart disease (6, 24, 25) or abnormal ECG (3, 5, 7, 26) is selected, probably because men have a higher prevalence of cardiovascular risk factors and other comorbidities. In our study, which included consecutive patients, 63% were men. Male patients had more diabetes and IHD. Moreover, RBBB was more frequent in men while LBBB morphology was more frequent in women. These findings in baseline characteristics are consistent with data previously published (3, 5, 7, 26–28), which suggests that patients included in the present study are likely representative of the population with syncope and BBB.

Paroxysmal aAVB is the most likely etiology of syncope in patients with BBB, but other causes also exist. In agreement with previous studies, we found that AVB is the mechanism of syncope in half of these patients, although significant differences were found between the sexes. Women less frequently had aAVB/sCD. In only 44.9% of women, compared to 55.0% of men, aAVB/sCD was found to be the cause of syncope, which represents a risk ratio of 0.81. In other words, women have a 19% lower risk of having aAVB/sCD. Even though there are some differences in patients' baseline characteristics, in multivariate analyses after adjusting for possible confounding variables, female sex was independently associated with a lower risk of advanced AVB (OR 0.44; 95% CI 0.26–0.74). Previous studies had shown that the risk of aAVB in the general population is higher in men (22, 29). For example, in a recent population-based cohort study, Kerola et al. (22) reported that male sex was an independent risk factor for the development of aAVB [adjusted HR 2.04 (95% CI 1.19–3.45)]. Thus, the present study reveals that these findings are also observed in patients with syncope and BBB and it is not explained by differences in the comorbidities alone.

It is well-known that women have a major susceptibility to reflex syncope (11–14). Moreover, previous studies have suggested that SND is also more prevalent in women (9, 10, 15). The higher prevalence of these etiologies in women observed in the general population is also applicable to patients with BBB and it may partially explain the relative lower rate of aAVB in these patients. In our study we only found small and not statistically significant differences in the incidence of these mechanisms between groups, probably because the study is underpowered. Moreover, it should be noted that some of these etiologies were usually diagnosed in Step 1 of the protocol that is not included in the analysis.

Interestingly, we found that the HV interval in the EPS was significantly longer in men. In particular, more men had an HV longer than 70 ms, suggesting that men have a more severe conduction disease. Despite these differences, EPS in women still offers a considerable diagnostic yield as has been previously reported (3, 8, 17), and even more importantly, NPV is similar between both sexes. In patients who were not diagnosed in Step 2, the use of an ICM offered a significant additional diagnostic yield in both groups. Remarkably, only a third of the diagnoses reached in Step 3 was due to aAVB. This finding supports the systematic use of an ICM in patients where EPS is not diagnostic.

Another key finding of the present study is that women have a lower risk of requiring a permanent pacemaker compared to men [adjusted HR 0.72 (95% CI 0.52–0.99)]. From the clinical point of view, this finding is especially relevant since pacemakers are useful to treat not only syncope due to aAVB/sCD but also due to other types of bradyarrhythmias and some cases of cardioinhibitory reflex syncope. Even though some of these bradyarrhythmias, such as sinus node dysfunction, seem to be more common in women, the overall risk of needing pacing is lower in women compared to men. Ahmed et al. investigated the predictors of pacemaker implantation in patients with syncope receiving an ICM (19). They found that female sex was an independent predictive factor for bradycardia necessitating pacemaker implantation. However, several differences are evident compared to our study. Firstly, only a quarter of the patients included had a BBB and EPS was not routinely performed. Second, less than of half of pacemakers were implanted due to AVB. SND was the most common indication for pacing and as has been commented previously, it seems to be more prevalent in women. Indeed, in the general population, pacemaker implantation is more common in men (9, 15, 29, 30). In a German registry of more than 17,000 patients with primary pacemaker implantation, 53% were men (29). In this large-scale patient cohort, it was found that male patients had more AV blocks when compared with women and less sick sinus syndrome and atrial fibrillation with bradycardia.

Although it was not the aim of the present study, it is remarkable that our results confirm that a systematic stepwise approach to evaluate syncope in patients with BBB, which was initially evaluated in the B4 study (3) and detailed in the ESC guidelines (1), is safe and achieves a high rate of etiological diagnosis allowing to select specific treatment and avoiding the implantation of unnecessary pacemakers. In the present study, once the diagnosis was reached and appropriately treated, only a few patients (8.9%) experienced a syncopal recurrence, most of them due to a vagal or orthostatic mechanism. This finding suggests that the diagnoses were specific. We also found that, compared to men, women had nearly half the mortality rate, probably in relation to a lower comorbidity burden (14).

The optimal management of patients with unexplained syncope and BBB is still controversial (1–4, 8, 18). In fact, the 2017 American College of Cardiology/the American Heart Association (ACC/AHA) guidelines (2) suggest empirical direct pacemaker implantation after exclusion of other syncope etiologies while ESC guidelines (1) recommend opting for a stepwise approach. In light of our results, gender may be an additional factor to be taken into account in the workup of patients with syncope and BBB. A stepwise approach seems reasonable to avoid unnecessary pacemaker implantation, especially in women, given that only half of them will require pacing because of the syncope. Nonetheless, randomized controlled trials are warranted to better answer this important question.

## Limitations

This study has certain limitations. It is an observational study carried out at a single high-volume center with a dedicated syncope clinic. To minimize potential biases inherent to the study's design, the patients were included consecutively, and possible confounding factors were analyzed. No genetic testing was done systematically to identify certain inherit heart disease that present a higher prevalence of sCD, however, the prevalence of these diseases is low. One aspect worth mentioning is that in our series, the prevalence of reflex/orthostatic syncope was low. It should be noted that some of these episodes were usually diagnosed in Step 1 of the protocol, prior to the patients' inclusion in the study. As such, this series refers not to the global etiology of syncope in this population, rather it focuses on those patients lacking an evident initial diagnosis. The study population was not ethnically diverse. All patients included in the study were from Caucasian or Latin, so the results observed may not be directly extrapolable to other ethnicities. Also, the tilt-test was not used in the workup protocol due to its low specificity in this population (1). However, in selected patients, tilt-test could have revealed an indication for pacing (1). Moreover, the study was not designed to assess predictors of pacemaker implantation in both groups.

## Conclusions

In this cohort study evaluating sex-specific differences in patients with unexplained syncope and BBB, we found that compared to men, women are at lower risk of having aAVB/sCD and of requiring cardiac pacing. A stepwise diagnostic approach based on EPS and long-term cardiac monitoring have similar diagnostic yield in both sexes and it seems appropriate to guide treatment and avoid unnecessary pacemaker implantation, especially in women.

## Data Availability Statement

The raw data supporting the conclusions of this article will be made available by the authors upon reasonable request.

## Ethics Statement

The studies involving human participants were reviewed and approved by Comitè d'Etica de Vall d'Hebron. Written informed consent for participation was not required for this study in accordance with the national legislation and the institutional requirements.

## Author Contributions

JF-P prepared the concept, design the study, performed statistical analysis, and draft and editing of the manuscript. NR-G participate in the study design, data review, and manuscript editing. IR-L prepared the clinical database and reviewed the study design. MB-O, CB-M, and MM-B recorded clinical data and revised data in the database. All authors contributed to design the manuscript, patients selection, manuscript review, and agreed with the content of its final version.

## Funding

This project was funded by ISCIII, CIBER, and Fundació Marató TV3 and co-funded by the European Regional Development Fund (ERDF-FEDER).

## Conflict of Interest

The Vall d'Hebron Arrhythmia Unit receives fellowship grants from Boston Scientific and Research grants from Abbott. JF-P receives advisory and speaking honoraria from Abbott and Microport. NR-G receives advisory and speaking honoraria from Abbott. BB, JP-R, and AS-O receive speaking honoraria from Abbott. The remaining authors declare that the research was conducted in the absence of any commercial or financial relationships that could be construed as a potential conflict of interest.

## Publisher's Note

All claims expressed in this article are solely those of the authors and do not necessarily represent those of their affiliated organizations, or those of the publisher, the editors and the reviewers. Any product that may be evaluated in this article, or claim that may be made by its manufacturer, is not guaranteed or endorsed by the publisher.

## Abbreviations

aAVB: Advanced atrioventricular block
sCD: Severe conduction disturbances
BBB: Bundle branch block
EPS: electrophysiological study
ICM: Implantable cardiac monitor
SND: Sinus node dysfunction.

## References

[1] Brignole M Moya A de Lange FJ Deharo JC Elliott PM Fanciulli A. ESC Guidelines for the diagnosis and management of syncope. Eur Heart J. (2018) 39:1883–948. 10.1093/eurheartj/ehy037 29562304

[2] Shen WK Sheldon RS Benditt DG Cohen MI Forman DE Goldberger ZD. ACC/AHA/HRS guideline for the evaluation and management of patients with syncope: a report of the American college of cardiology/American Heart Association task force on clinical practice guidelines and the Heart Rhythm Society. Circulation. (2017). 136:e60–122. 10.1161/CIR.0000000000000499 28280231

[3] Moya A García-Civera R Croci F Menozzi C Brugada J Ammirati F. Diagnosis, management, and outcomes of patients with syncope and bundle branch block. Eur Heart J. (2011) 32:1535–41. 10.1093/eurheartj/ehr071 21444367 PMC3114095

[4] Roca-Luque I Francisco-Pascual J Oristrell G Rodríguez-García J Santos-Ortega A Martin-Sanchez G. Syncope, conduction disturbance, and negative electrophysiological test: Predictive factors and risk score to predict pacemaker implantation during follow-up. Heart Rhythm. (2019) 16:905–12. 10.1016/j.hrthm.2018.12.015 30576876

[5] Roca-Luque I Oristrell G Francisco-Pasqual J Rodríguez-García J Santos-Ortega A Martin-Sanchez G. Predictors of positive electrophysiological study in patients with syncope and bundle branch block: PR interval and type of conduction disturbance. Clin Cardiol. (2018) 41:1537–42. 10.1002/clc.23079 30251426 PMC6489853

[6] Francisco-Pascual J Rodenas E Rivas-Gándara N Belahnech Y San Emeterio AO Pérez-Rodón J. Etiology and prognosis of patients with unexplained syncope and mid-range left ventricular dysfunction. Heart Rhythm. (2020) 18:597–604. 10.1016/j.hrthm.2020.12.009 33326869

[7] Martí-Almor J Cladellas M Bazán V Delclós J Altaba C Guijo MA. Novel predictors of progression of atrioventricular block in patients with chronic bifascicular block. Rev Española de Cardiol. (2010) 63:400–8. 10.1016/S1885-5857(10)70088-8 20334805

[8] Sheldon RS Lei LY Solbiati M Chew DS Raj SR Costantino G. Electrophysiology studies for predicting atrioventricular block in patients with syncope: a systematic review and meta-analysis. Heart Rhythm. (2021) 18:1310–7. 10.1016/j.hrthm.2021.04.010 33887450

[9] Linde C Bongiorni MG Birgersdotter-Green U Curtis AB Deisenhofer I Furokawa T. Sex differences in cardiac arrhythmia: a consensus document of the European Heart Rhythm Association, endorsed by the Heart Rhythm Society and Asia Pacific Heart Rhythm Society. EP Eur. (2018) 20:1565. 10.1093/europace/euy067 29961863

[10] Ehdaie A Cingolani E Shehata M Wang X Curtis AB Chugh SS. Sex differences in cardiac arrhythmias. Circulation. (2018) 11:5680. 10.1161/CIRCEP.117.005680 29874167

[11] Romme JJCM van Dijk N Boer KR Dekker LRC Stam J Reitsma JB. Influence of age and gender on the occurrence and presentation of reflex syncope. Clin Autonomic Res. (2008) 18:127–33. 10.1007/s10286-008-0465-0 18449594

[12] Park J Jang SY Yim HR On On YK Huh J Shin D-H. Gender difference in patients with recurrent neurally mediated syncope. Yonsei Med J. (2010) 51:499–503. 10.3349/ymj.2010.51.4.499 20499413 PMC2880260

[13] Deveau AP Sheldon R Maxey C Ritchie D Doucette S Parkash R. Sex differences in vasovagal syncope: a post hoc analysis of the Prevention of Syncope Trials (POST) I and II. Can J Cardiol. (2020) 36:79–83. 10.1016/j.cjca.2019.10.008 31810744

[14] Bernier R Tran DT Sheldon RS Kaul P Sandhu RK. A population-based study evaluating sex differences in patients presenting to emergency departments with syncope. JACC Clin Electrophysiol. (2020) 6:341–7. 10.1016/j.jacep.2019.11.002 32192686

[15] Bernal O Moro C. Cardiac arrhythmias in women. Rev Española de Cardiol. (2006) 59:609–18. 10.1016/S1885-5857(07)60011-5 16790203

[16] Kusumoto FM Schoenfeld MH Barrett C Edgerton JR Ellenbogen KA Gold MR ACC/AHA/HRS ACC/AHA/HRS Guideline on the Evaluation and Management of Patients With Bradycardia and Cardiac Conduction Delay: a Report of the American College of Cardiology/American Heart Association Task Force on Clinical Practice Guidelines and the Heart Rhythm Society. Circulation. (2019). 140:e382–482. 10.1161/CIR.0000000000000627 30586772

[17] Roca-Luque I Francisco-Pasqual J Oristrell G Rodríguez-García J Santos-Ortega A Martin-Sanchez G. Flecainide versus procainamide in electrophysiological study in patients with syncope and wide QRS duration. JACC Clin Electrophysiol. (2019) 5:212–9. 10.1016/j.jacep.2018.09.015 30784693

[18] Moya A Rivas-Gandara N Perez-Rodón J Franciso-Pascual J Santos-Ortega A Fumero P. Syncope and bundle branch block: diagnostic approach. Herzschrittmacherther Elektrophysiol. (2018) 29:161–5. 10.1007/s00399-018-0560-4 29696347

[19] Ahmed N Frontera A Carpenter A Cataldo S Connolly GM Fasiolo M. Clinical predictors of pacemaker implantation in patients with syncope receiving implantable loop recorder with or without ECG conduction abnormalities. Pacing Clin Electrophysiol. (2015) 38:934–41. 10.1111/pace.12666 25973599

[20] Francisco-Pascual J Olivella San Emeterio A Rivas-Gándara N Pérez-Rodón J Benito B Santos-Ortega A. High incidence of subclinical atrial fibrillation in patients with syncope monitored with implantable cardiac monitor. Int J Cardiol. (2020) 316:110–6. 10.1016/j.ijcard.2020.05.078 32470530

[21] Fedorowski A Pirouzifard M Sundquist J Sundquist K Sutton R Zöller B. Risk factors for syncope associated with multigenerational relatives with a history of syncope. JAMA Netw Open. (2021) 4:e212521. 10.1001/jamanetworkopen.2021.2521 33783519 PMC8010588

[22] Kerola T Eranti A Aro AL Haukilahti MA Holkeri A Junttila MJ. Risk factors associated with atrioventricular block. JAMA Netw Open. (2019) 2:e194176. 10.1001/jamanetworkopen.2019.4176 31125096 PMC6632153

[23] Soteriades ES Evans JC Larson MG Chen MH Chen L Benjamin EJ. Incidence and prognosis of syncope. N Engl J Med. (2002) 347:878–85. 10.1056/NEJMoa012407 12239256

[24] Francisco-Pascual J Rodenas E Belahnech Y Rivas-Gándara N Pérez-Rodon J Santos-Ortega A. Syncope in patients with severe aortic stenosis: more than just an obstruction issue. Can J Cardiol. (2021) 37:284–91. 10.1016/j.cjca.2020.04.047 32439473

[25] Shenthar J Prabhu MA Banavalikar B Benditt DG Padmanabhan D. Etiology and outcomes of syncope in patients with structural heart disease and negative electrophysiology study. JACC Clin Electrophysiol. (2019) 2019:871. 10.1016/j.jacep.2019.01.021 31122384

[26] Azocar D Ruiz-Granell R Ferrero A Martínez–Brotons Á Izquierdo M Domínguez E. Syncope and bundle branch block. Diagnostic yield of a stepped use of electrophysiology study and implantable loop recorders. Rev Española de Cardiol. (2011) 64:213–9. 10.1016/j.rec.2010.10.017 21330036

[27] Rasmussen PV Skov MW Ghouse J Pietersen A Hansen SM Top-Pedersen C. Clinical implications of electrocardiographic bundle branch block in primary care. Heart. (2019) 105:1160–7. 10.1136/heartjnl-2018-314295 31129608

[28] Bussink BE van Ginhoven TM Smit PC. Right bundle branch block: prevalence, risk factors, and outcome in the general population: results from the Copenhagen City Heart Study. Eur Heart J. (2013) 34:138–46. 10.1093/eurheartj/ehs291 22947613

[29] Nowak B Misselwitz B Erdogan A Funck R Irnich W Israel CW. Do gender differences exist in pacemaker implantation?–results of an obligatory external quality control program. Europace. (2010) 12:210–5. 10.1093/europace/eup312 19864309

[30] Kataoka S Kobayashi Y Isogai T Tanno K Fukamizu S Watanabe N. Permanent pacemaker implantation and its predictors in patients admitted for complete atrioventricular block: a report from the Tokyo Cardiovascular Care Unit Network multi-center registry. Heart Vessels. (2020) 35:1573–82. 10.1007/s00380-020-01642-9 32500173

