# Supplementary material for: Sex-Related Differences in Patients With Unexplained Syncope and Bundle Branch Block: Lower Risk of AV Block and Lesser Need for Cardiac Pacing in Women
Source: Front Cardiovasc Med. 2022 Feb 25;9:838473. doi: 10.3389/fcvm.2022.838473 (PMC8914040; doi:10.3389/fcvm.2022.838473)
Supplement: Supplementary file 1 [file Data_Sheet_1.PDF]

SUPPLEMENTARY FILES:

Supplementary Table S1

Definitions of etiology of syncope

**Syncope Diagnosis[1]**	**Definition**	
Vasovagal Syncope	**Certain**	Precipitated by emotional distress or orthostatic stress and associated with typical prodrome
	**Highly Probable**	Associated with typical prodrome but without clear trigger, or associated with typical trigger with minimal prodrome<br><br>Or<br><br>ECG recording in the ICM suggestive of reflex origin (Type 1A, 1B,2 of the ISSUE classification)
Carotid sinus hypersensitivity	**Certain**	Syncope is reproduced in the presence of asystole > 3 seg and/or fall in systolic BP>50 mmHg during CSM
	**Highly Probable**	presence of asystole > 3 seg and/or fall in systolic BP > 50 mmHg during CSM without reproducing the syncope
Orthostatic Syncope	**Certain**	Occurs after standing up and there is documentation of symptomatic orthostatic hypotension (OH) in active standing test
	**Highly Probable**	Occurs after standing up and there is documentation of asymptomatic OH in active standing test.<br><br>Or<br><br>Occurs after standing and the ECG recording in the ICM is compatible with OH response (Type 3 or Type 4A of ISSUE classification)
Arrhythmic Syncope	**Certain**	Clear correlation between arrhythmic event and syncope, or diagnostic findings in the EPS: HV interval ≥ 70 msec or ≥100 msec after class I drug challenge, Advanced AV block, induction of VT or SVT that reproduces the symptoms.
	**Highly Probable**	Documentation of asymptomatic advanced AV block, asystole > 3 secs (except asleep), SVT or VT. SNRTc > 525 ms in the EPS+ documented sinus pauses on ECG monitoring.
Advanced AVB / Severe conduction disturbances	**Certain**	Correlation between type II 2nd degree, 3d degree or high-grade AVB event and

		syncope, or diagnostic findings in the EPS: HV interval ≥ 70 msec or ≥100 msec after class I drug challenge, intra-Hisian or infra-Hisian block
	**Highly Probable**	Documentation of asymptomatic . type II 2nd degree, 3d degree or high-grade AVB
Sinus node disfunction	**Certain**	Clear correlation between sinus pause and syncope.
	**Highly Probable**	SNRTc > 525 ms in the EPS+ documented sinus pauses on ECG monitoring. Asystole > 3 secs (except asleep).
Acute coronary syndrome	**Highly Probable**	Occurs during the acute phase of an ACS, with typical chest pain, acute ischemic ECG changes and/or troponin rise.
Exercise Low Cardiac Output	**Highly Probable**	Occurs during intense exercise such as fast walking, running or dancing in presence of a ventricular outflow obstruction (like aortic stenosis), without criteria for previous diagnosis

CSM: carotid sinus massage / BP: blood pressure / OH: orthostatic hypotension / EPS: Electrophysiology study / AV: atrio-ventricular / VT: ventricular tachycardia / SVT: Supraventricular tachycardia / SNRTc: corrected sinus node recovery time / ACS: acute coronary syndrome

TABLE S2 –Logistic regression model to assess the association between sex and aAVB/sCD.

	Factor	*OR*	*OR 95% CI*	*p value*
Unadjusted				
	Women	***0.66***	***0.45-0.98***	***0.038***
Adjusted				
	Women	***0.44***	***0.26-0.74***	***0.002***
	Age>75 y.o	*1.13*	*0.70-1.84*	*0.618*
	Hypertension	*1.07*	*0.59-1.92*	*0.834*
	Diabetes	*0.94*	*0.57-1.55*	*0.805*
	IHD	*1.20*	*0.68-2.13*	*0.553*
	LVEF<45%	*0.79*	*0.40-1.57*	*0.498*
	Atrial Fibrillation	*1.03*	*0.48-2.22*	*0.942*
	Recurrent syncope	*1.21*	*0.75-1.94*	*0.430*
	LBBB	*2.80*	*1.21-6.47*	*0.016*
	Isolated RBBB	*0.29*	*0.09-0.89*	*0.030*
	RBBB and LAFH	*0.89*	*0.41-1.93*	*0.773*
	Long PR interval	*2.52*	*1.48-4.27*	*0.001*

CI: confidence interval; OR: odds ratio; y.o: years old. IHD: Ischemic heart disease. LVEF: Left ventricular ejection fraction; LBBB: Left bundle branch block; RBBB: Right bundle branch block; LAFB: Left anterior fascicular block.

TABLE S3 **Type of cardiac device implanted to treat the syncope**

**Type of device**	**TOTAL**	**MEN**	**WOMEN**
	**(n=252)**	**(n=167)**	**(n=85)**
VVI pacemaker, *n %*	82 (32.5)	51 (30.5)	31 (36.5)
DDD pacemaker, *n %*	155 (61.2)	103 (61.7)	52 (61.2)
CRT-pacemaker, *n %*	7 (2.8)	7 (4.2)	0 (0)
Single chamber ICD, *n %*	2 (0.8)	2 (1.2)	0 (0)
Dual chamber ICD, *n %*	2 (0.8)	2 (1.2)	0 (0)
CRT-ICD, *n %*	4 (2.4)	2 (1.2)	2 (2.4)

CRT: Cardiac resynchronization therapy. ICD: Implantable cardiac defibrillator

TABLE S4 – Mechanism of syncope recurrence after the diagnosis and appropriate treatment.

**Mechanism**	**TOTAL**	**MEN**	**WOMEN**
	**(n=30)**	**(n=19)**	**(n=11)**
Orthostatic	16 (53.3)	9 (47.4)	7 (63.6)
Reflex	2 (6.7)	2 (10.5)	0 (0)
aAVB/sCD	2 (6.7)	2 (10.5)	0 (0)
Low cardiac output	1 (3.3)	0 (0)	1 (9.1)
VT	2 (6.7)	2 (10.5)	0 (0)
Fast SVT/AF	2 (6.7)	1 (5.3)	1 (9.1)
SND	0	0	0
CSH	0	0	0
Pacemaker dysfunction	2 (6.7)	1 (5.3)	1 (9.1)
Other	1 (3.3)	1 (5.3)	0 (0)
Unknown, n %	2 (6.7)	1 (5.3)	1 (9.1)

aAVB/sCD: advanced atrio-ventricular block or severe conduction disturbances; VT: Ventricular Tachycardia; SND: Sinus node disfunction. SVT: Supraventricular tachycardia. AF: Atrial tachycardia. CSH: Carotid sinus hypersensitivity
